# Barriers and facilitators for online genetic care for hereditary cancer in Japan: findings from surveys of both clients and medical professionals

**DOI:** 10.1007/s10147-026-03026-x

**Published:** 2026-04-21

**Authors:** Sayaka Ueno, Yusaku Urakawa, Fumino Kato, Arisa Ueki, Keika Kaneko, Koji Matsumoto, Hiromi Sugawara, Sayoko Takeuchi, Reiko Yoshida, Miho Kakuta, Kiwamu Akagi, Kazuo Tamura, Akira Hirasawa

**Affiliations:** 1 Department of Genomic Medicine, School of Medicine, Fujita Health University, Aichi, Japan; 2 Department of Clinical Genomic Medicine, Okayama University Graduate School of Medicine, Dentistry and Pharmaceutical Sciences, Okayama, Japan; 3 Division of Clinical Genetic Oncology, Cancer Institute Hospital, Japanese Foundation for Cancer Research, Tokyo, Japan; 4 Division of Clinical Genetics, Hyogo Cancer Center, Hyogo, Japan; 5 Department of Medical Genetics and Genomics, Saitama Cancer Center, Saitama, Japan; 6 Center for Genomic Diagnosis, International University of Health and Welfare (IUHW) Narita Hospital, Chiba, Japan; 7 Division of Genetic Medicine, Master of Science, Graduate School of Science and Engineering Research, Kindai University, Osaka, Japan

**Keywords:** Hereditary cancer, Remote medical care, Barriers to online genetic care, Facilitators for online genetic care

## Abstract

**Background:**

Online genetic care can offer a promising solution to the shortage of qualified medical professionals in genetic medicine, which leads to regional disparities in access. However, despite global adoption, its use in Japan remains limited.

**Methods:**

Two questionnaire surveys were conducted to investigate potential needs and barriers regarding online genetic care: one involving 858 medical professionals (738 physicians and 120 genetic counselors or nurses), and the other involving 443 clients who received in-person genetic counseling.

**Results:**

Only 14.0% of the medical professionals had experience with online genetic care, although 85.9% of the professionals engaged in cancer genetics were willing to consider providing it in the future. Notably, a discrepancy was found regarding hospital selection: clients prioritized access to specialized medical care, whereas professionals assumed clients valued accessibility for family members. Professionals expressed greater concerns about adequacy of online communication, client environments and internet security. Among clients, 89.1% estimated they would sufficiently understand and accept total content of counseling session if were conducted online. Older age and infrequent internet use were associated with lower acceptance and higher anxiety regarding online methods. Concerns about ability to use the necessary technology affected clients’ willingness to encourage online care for their relatives.

**Conclusion:**

Online genetic care shows high potential for client acceptance and can effectively address regional disparities in Japan. To bridge the gap between client needs and professional perceptions and to overcome the digital divide, it is necessary to develop secure, accessible systems and provide education for both patients and healthcare providers.

## Introduction

In cancer treatment, genetic information is important in selecting appropriate surgical procedures, drug treatments, and preventive measures [1–4]. Despite rapidly growing demand for genetic care, access to it is hampered by a significant shortage of qualified medical professionals, leading to regional disparities. Several studies in North America and Europe have demonstrated that remote genetic counselling (GC) is noninferior to in-person visits [5–7]. The pandemic accelerated the global adoption of online GC [8–10]. Consequently, many reports have emerged on the use of online genetic practice in various clinical settings. Bergstrom et al. found that many of genetic counselors felt satisfied with remote GC via video or telephone [11]. Pereira et al. reported that all clients who had received video GC wished to receive such counseling again, with half preferring this format to an in-person appointment [12].

One advantage of online GC is that blood relatives in distant locations can also participate via the Internet. To date, attempts have been made to conduct cascade testing for at-risk relatives by telephone or online. Frey et al. conducted telephone GC by directly contacting at-risk relatives with the consent of probands with newly diagnosed cancer-associated pathogenic variants [13]. In that study, 69.5% of relatives underwent genetic testing, the psychological burden was small, and a high level of satisfaction was obtained at the time of testing and 6 months later.

Despite these developments and the introduction of insurance coverage for online medical care postpandemic, their adoption in Japan remains low. A survey by Sugawara et al. reported that only 5.29% of respondents had experience with online medical care [14]. Even more limited would be online genetic care in Asian countries, including Japan. Only a few studies have investigated patients’ preference regarding online GC. Nishiyama et al. reported that, when presented with multiple GC models, including online GC, 80.7% of pregnant women undergoing prenatal testing preferred face-to-face GC, with only 2.6% preferring online GC [15]. Conversely, Sim et al.’s survey of 160 cancer patients in Singapore who had already undergone genetic testing and knew their results revealed that most participants were open to receiving GC via phone (71.3%) or video (66.3%) [16].

Our study has two objectives: to determine the frequency of taking part in remote practice among genetic medical professionals and to identify barriers to and potential needs for online genetic care, including GC, genetic testing and surveillance in Japan.

## Methods

### Study design

**Fig. 1:**
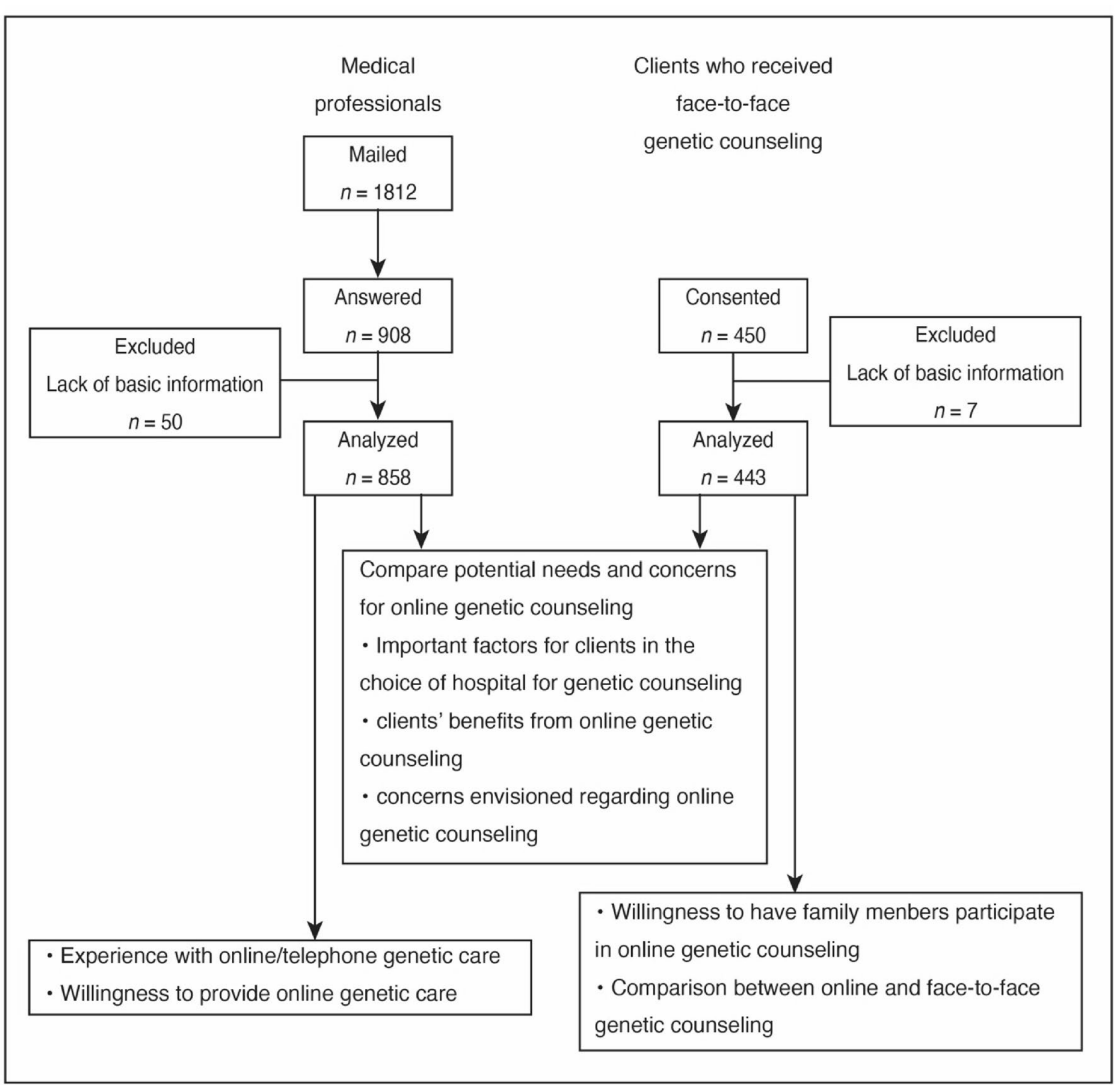
Study design and participants’ flow diagram

We conducted two questionnaire surveys: one for clients receiving genetic care for hereditary cancer and one for medical professionals providing genetic care. This study was approved by the Okayama University Review Board (protocol numbers 2202-048 and 2202-054). Figure 1 shows a flow diagram of the study design and participant recruitment.

### Participants

Medical professionals were eligible if they were a medical doctor certified as clinical geneticist or hereditary oncology specialist, certified genetic counsellor (CGC), or certificated nurse specialist (CNS) in genetic nursing. The analysis included responses from filling out the survey form on paper or online between July and December 2022.

Clients were eligible if they were ≥ 18 years old, had received in-person GC about hereditary cancer syndrome, could communicate at a conversational level in Japanese, and were capable of independent decision-making. Recruitment was coordinated among five hospitals (Okayama University Hospital, The Cancer Institute Hospital of JFCR, Saitama Cancer Center, Showa University Hospital, and Hyogo Cancer Center). All are leading centers of hereditary cancer medicine in Japan, providing GC for more than 200 hereditary cancer cases per year. Two hospitals (The Cancer Institute Hospital of JFCR and Showa University Hospital) are in central Tokyo, while the remaining three are in slightly more rural areas. The survey was conducted from July 2022 to December 2023.

### Questionnaire development

The primary objective was to investigate potential need for online genetic care. For the client group, we inquired about (1) time taken for hospital visits, (2) important factors when choosing a hospital for GC (five-point scale), (3) benefits envisioned for online GC (five-point scale), and (4) willingness to have their family members participate in online GC (five-point scale). We also asked them to estimate their level of understanding and acceptance if their recent GC had been conducted online. For medical professionals, we inquired about (1) important factors in the client’s choice of a hospital for GC (five-point scale), (2) experience with online/telephone genetic care both for clients and family members, (3) clients’ benefits from online GC (five-point scale), and (4) benefits of online GC for the broader practice of genetic medicine (five-point scale).

The secondary objective was to identify barriers toward online GC. Both groups were asked about their experience using web conferencing tools and concerns regarding online GC. Clients were also asked about the devices they use to access the internet.

### Data analysis

Fisher’s exact test was used for binary variables and Mann–Whitney U test for categorical variables. A multivariate logistic regression analysis was performed for the outcomes regarding experience and willingness of online genetic care among medical professionals. We chose variables with *p* < 0.1 for further multivariate analysis. We set *p* < 0.05 as statistically significant. All analyses were performed using Prism version 10 (GraphPad Software, Boston, MA) or Stata version 17.0 (StataCorp LLC, College Station, TX).

## Results

### Participant characteristics

We mailed questionnaires to 1812 medical professionals and received responses from 908 (50.1%). Of these, 858 who provided complete basic information (gender, age, profession, and years engaged in genetic medicine) were included in the final analysis. As shown in Table 1, 86.0% (738/858) were medical doctors and 14.0% (120/858) were CGCs or CNSs, 59.7% (512/858) were engaged in cancer genetics, and 49.4% (424/858) were “confident” or “generally comfortable” with hosting a web conference.

**Table 1:** Background of medical professional survey respondents

*Gender*	
Male	441
Female	408
Prefer not to say	9
*Age*	
< 40	148
40 s	352
50 s	247
≥ 60	111
*Profession*	
Medical doctor	738
CGC	111
CNS	9
*Experience (years)*	
< 5	160
5–9	268
10–14	202
15–19	81
≥ 20	147
*Become a web conference host*	
Confident	134
Generally comfortable	290
Neither confident nor anxious	142
Slightly anxious	158
Very anxious	117
N/A	17
*Engaged in cancer genetics*	
Yes	512
No	343
N/A	3

A total of 450 clients agreed to participate and completed the survey. The characteristics of the 443 participants who provided gender and age information are shown in Table 2. Among the participants, 13 (2.9%) did not use the Internet. For 33.0% (146/443), traveling to the hospital for GC took over 60 min. The survey was conducted at the time of pretest GC for about half of the clients.

**Table 2:** Client characteristics

*Gender*	
Male	87
Female	356
*Age*	
20 s	48
30 s	58
40 s	128
50 s	109
60 s	66
≥ 70	34
Median (range)	49.0 (20–80)
*Internet use*	
None	13
Only at the office	7
Only at home	164
Both at home and office	255
N/A	4
*Purpose of visit*	
Pretest counseling	203
Posttest counseling	114
Follow-up counseling	126
*Time required for hospital visit (minutes)*	
≤ 30	110
31– ≤ 60	195
61– ≤ 90	85
91– ≤ 120	29
> 120	32

### Important factors for clients in selecting a hospital for genetic care

**Fig. 2:**
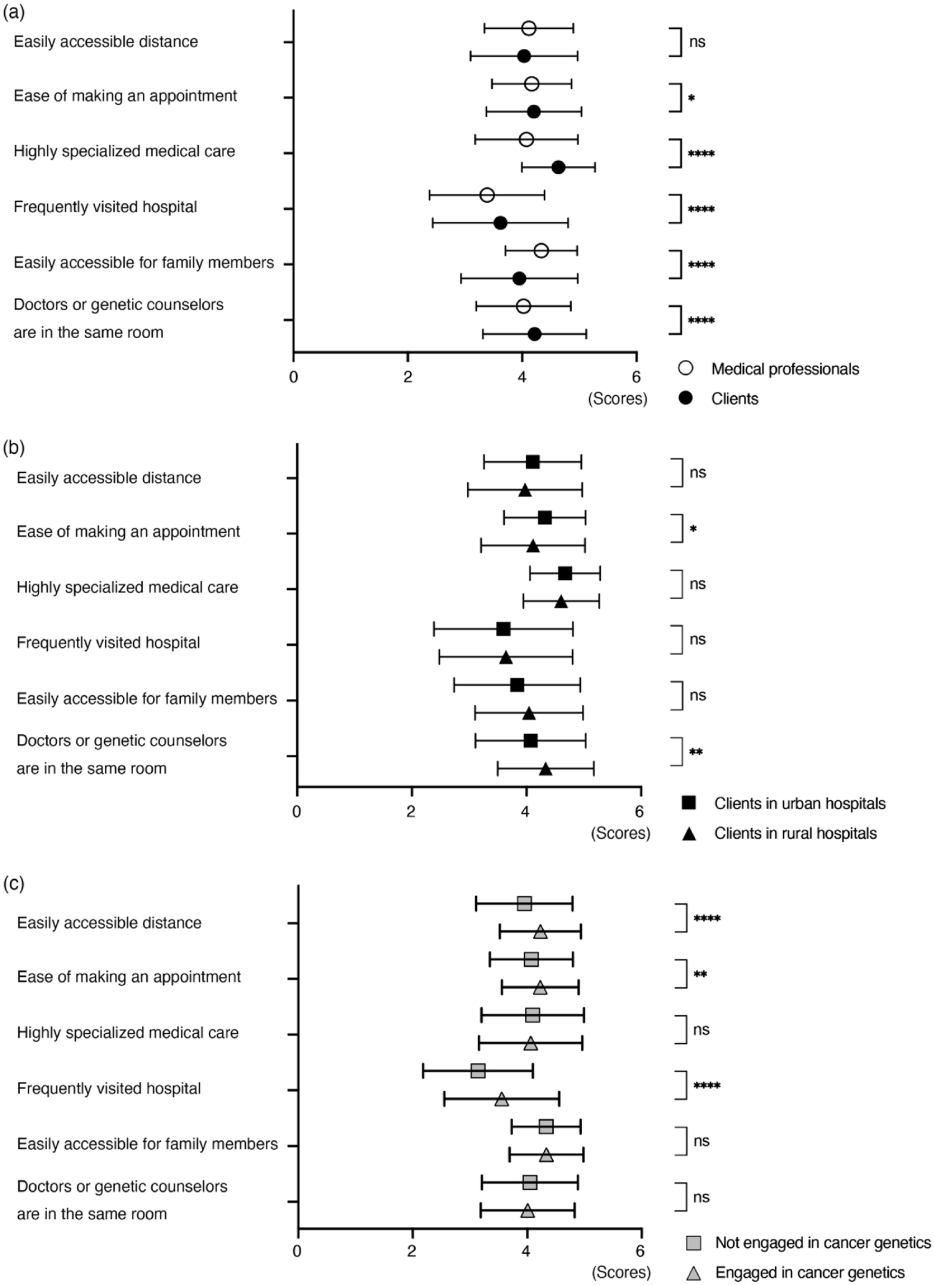
Important points in selecting a hospital for genetic medical care. **a** The most important factor for clients was “Highly specialized medical care,” which was also the factor with the largest difference from the medical professionals’ ratings. Among the medical professionals, “Easily accessible for family members” was rated highest. **b** Clients at urban hospitals rated ease of making appointments more highly, whereas those at rural hospitals rated face-to-face communication more highly. **c** Medical professionals currently engaged in cancer genetics perceived easier access to and familiarity with medical facilities to be important factors for clients. Results from medical professionals (*n* = 857) and clients (*n* = 441) are shown (mean with standard deviation). *p < 0.05, **p < 0.01, ***p < 0.001, ****p < 0.001

Both groups were asked about factors important for clients in selecting a hospital for genetic care (Fig. 2a). While no significant difference was found in the perceived importance of accessibility in terms of distance, clients placed greater importance on other factors than medical professionals did, with the exception of “Easy accessibility for family members.” The most important factor for clients was “Highly specialized medical care,” which also showed largest discrepancy with the medical professionals’ ratings. Among the medical professionals, “Easily accessible for family members” was rated highest.

A comparison between clients who visited urban and rural hospitals revealed that the former prioritized “ease of making appointments” while placing less emphasis on face-to-face GC (Fig. 2b). Medical professionals currently engaged in cancer genetics assumed that clients prioritized accessibility and familiarity with the medical institution when receiving genetic care (Fig. 2c).

### Assumed benefits and concerns regarding online GC

**Fig. 3:**
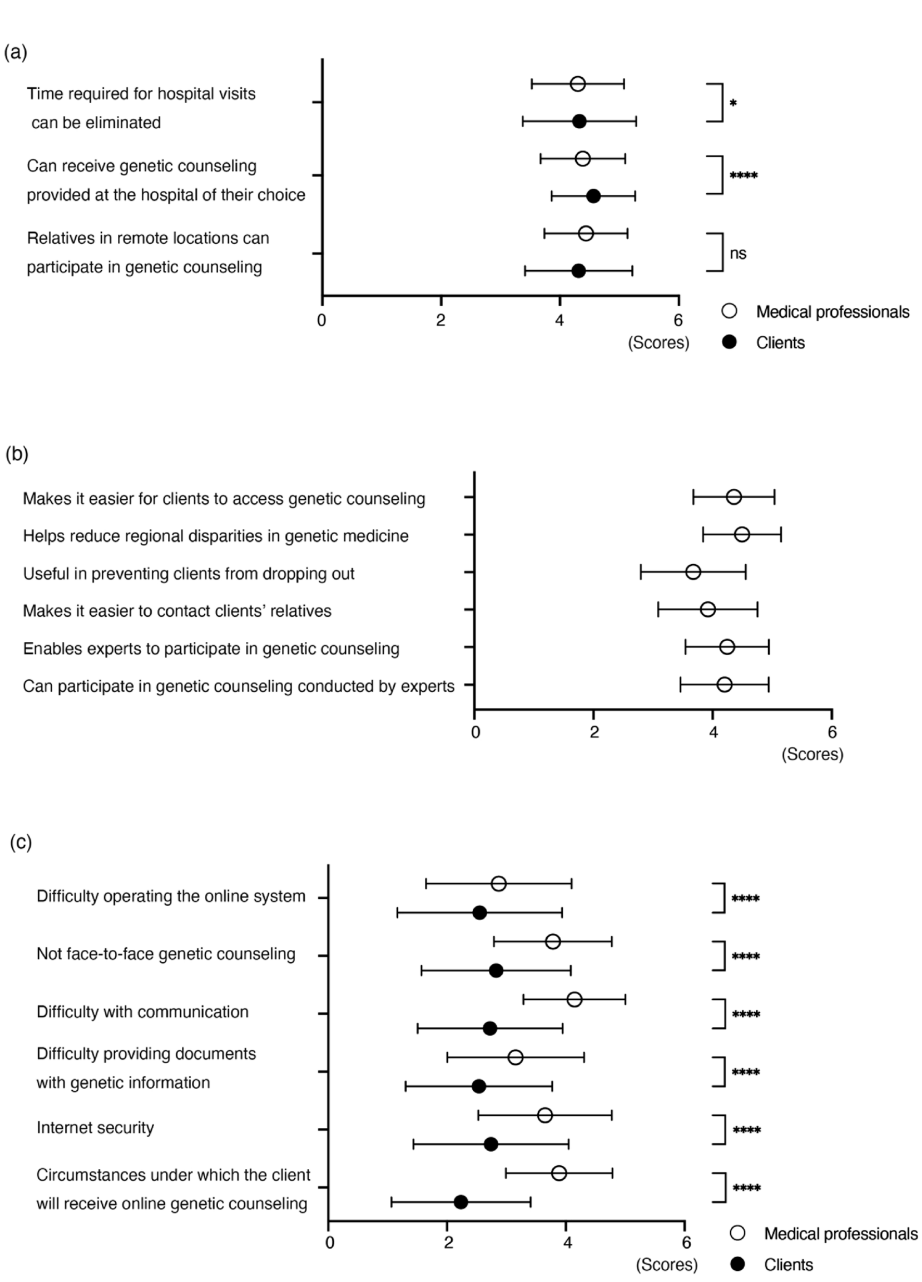
Possible benefits and concerns regarding online genetic counselling. **a** Clients felt that they most benefited from being able to choose the hospital. **b** Medical professionals felt that there were strong benefits in clients’ access and reducing regional disparities. **c** Medical professionals had greater concerns about online genetic care than clients did, especially regarding “Difficulty with communication” and “Circumstances under which the clients will receive online genetic counseling.” Results from medical professionals (*n* = 857) and clients (n = 441) are shown (mean with standard deviation). *p < 0.05, **p < 0.01, ***p < 0.001, ****p < 0.001

Both groups were asked about the possible benefits and concerns about online GC. The medical professionals were also asked about the extent to which such care would be helpful in promoting and improving genetic care. Both the medical professionals and clients felt online GC would have strong benefits for clients (Fig. 3). Notably, clients perceived greater benefits for being able to receive genetic care at their hospital of choice. Clients generally had less concerns about online GC than the medical professionals. Concerns about the online medical care system’s operation were similar between the two groups, though the medical professionals had greater concerns about the quality of online communication. Regarding the perceived benefits and concerns about online genetic care, no significant influence was observed based on whether the medical professional was currently engaged in cancer genetics, except for questions related to clients’ relatives; “Relatives in remote locations can participate in GC” [4.50 (95% CI: 4.44–4.55) vs. 4.35 (4.27–4.43)], and “Makes it easier to contact client’ relatives” [4.02 (3.95–4.08) vs. 3.79 (3.70–3.88)].

### Factors associated with medical professionals’ experience and willingness

Of the 853 medical professionals, 120 (14.0%) had experience with online genetic care (Table 3). Among them, 70.8% (85/120) were “confident” or “generally comfortable” with hosting a web conference. Regardless of whether or not they had experience with online genetic care, medical professionals assumed a relatively high level of benefits for clients. Being a CGC or CNS, having ≥ 10 years of experience, confident in being a web conference host, and engaged in cancer genetics were positively associated with experience in online genetic care. Greater concern about such care was negatively associated with experience.

**Table 3:** Multivariate analysis of factors related to online genetic counseling experience and willingness to provide such counseling in the future

Factors	Experience		*p*	Adjusted odds ratio (95% confidence interval)
	Yes (*n* = 120)	No (*n* = 733)		
*Gender*				
Male	65	374		
Female	53	352	0.4882	
Prefer not to say	2	7		
*Age*				
< 50	61	437		
≥ 50	59	296	0.1161	
*Profession*				
Medical doctor	92	642		
Genetic counselor, CNS	28	91	0.0026	2.18 (1.30–3.65)
*Experience (years)*				
< 10	48	379		
≥ 10	72	354	0.0182	2.10 (1.36–3.27)
*Become a web conference host*				
Neutral to very anxious	34	380		
Confident/generally comfortable	85	337	< 0.0001	2.00 (1.26–3.19)
N/A	1	16		
*Engaged in cancer genetics*				
No	32	308		
Yes	86	424	0.0023	2.00 (1.26–3.18)
N/A	2	1		
Benefits for clients	4.36 ± 0.62	4.38 ± 0.58	0.9921	
Benefits in genetic medicine practice	4.23 ± 0.60	4.14 ± 0.55	0.0349	1.21 (0.82–1.77)
Concerns	3.29 ± 0.82	3.63 ± 0.66	< 0.0001	0.62 (0.46–0.84)

Factors	Willingness		*p*	Adjusted odds ratio (95% confidence interval)
	Yes (*n* = 440)	No (*n* = 72)		
*Gender*				
Male	216	41		
Female	217	30	0.2496	
*Prefer not to say*	7	1		
Age				
< 50	276	37		
≥ 50	164	35	0.0696	0.73 (0.43–1.23)
*Profession*				
Medical doctor	349	63		
Genetic counselor, nurse	91	9	0.1114	
*Experience (years)*				
< 10	248	38		
≥ 10	192	34	0.6095	
*Become a web conference host*				
Neutral to very anxious	199	42		
Confident/generally comfortable	233	27	0.0271	1.32 (0.76–2.31)
N/A	8	3		
Benefits for clients	4.42 ± 0.54	4.19 ± 0.75	0.0279	1.16 (0.71–1.91)
Benefits in genetic medicine practice	4.23 ± 0.52	3.90 ± 0.63	< 0.0001	2.33 (1.34–4.08)
Concerns	3.54 ± 0.70	3.90 ± 0.63	0.0001	0.56 (0.36–0.86)

Among medical professionals engaged in cancer genetics, 85.9% (440/512) indicated a willingness to provide online genetic care in the future. A multivariate analysis showed that this willingness was positively correlated with the perceived benefits for genetic medicine and negatively correlated with concerns about online genetic care. Confidence in hosting a web conference was not significantly associated with future willingness.

### Comparison of face-to-face and online medical care for clients

After face-to-face GC, clients were asked to estimate their understanding and acceptance if the counseling session was conducted online. Among them, 384 (89.1%) indicated that they would understand and willingly accept at least 70–80% of the session’s content (Table 4). The remaining 10.9% felt their understanding would be half or less. Univariate analysis showed that assumed higher level of understanding and acceptance was associated with younger age (< 50 years), having both work and personal Internet use, and perception of greater benefits and lesser concerns with online care.

**Table 4:** Univariate analysis of factors associated with assumed level of understanding or extent of encouragement for relatives

Factors	Assumed levels of understanding			Extent of encouragement			
	≥ 70–80%	≤ 50%		Will encourage	Depends on conditions	Not willing to encourage	
	(*n* = 384)	(*n* = 57)	*p*	(*n* = 139)	(*n* = 276)	(*n* = 27)	*p*
*Gender*							
Male	78	8		38	46	2	
Female	306	49	0.3695	101	230	25	0.0113
*Age*							
< 50	213	21		72	151	11	
≥ 50	171	36	0.0102	67	125	16	0.3648
*Purpose of visit*							
Before genetic testing	192	31		67	123	13	
Disclosure of results	104	10		40	66	8	
Follow-up	108	16	0.3997	32	87	6	0.3931
*Locations using internet*							
Both office and home	230	24		80	161	14	
Others	151	32	0.0196	59	113	11	0.9329
N/A	3	1		0	2	2	
Benefits for clients	4.47 ± 0.61	3.96 ± 0.83	< 0.0001	4.69 ± 0.49	4.33 ± 0.63	3.69 ± 1.00	< 0.0001
Concerns	2.47 ± 0.86	3.54 ± 0.91	< 0.0001	2.30 ± 0.99	2.70 ± 0.86	3.20 ± 1.05	< 0.0001

When asked if they would encourage relatives to attend a GC session using a web conferencing tool, 139 (31.4%) answered yes, while 276 (62.4%) answered it would depend on the conditions and 27 (6.1%) answered no. The extent of encouragement was positively correlated with male gender and greater perceived benefits for online genetic care and negatively correlated with concerns about online genetic care.

### Computing devices usage and experience with video conferencing tools

**Fig. 4:**
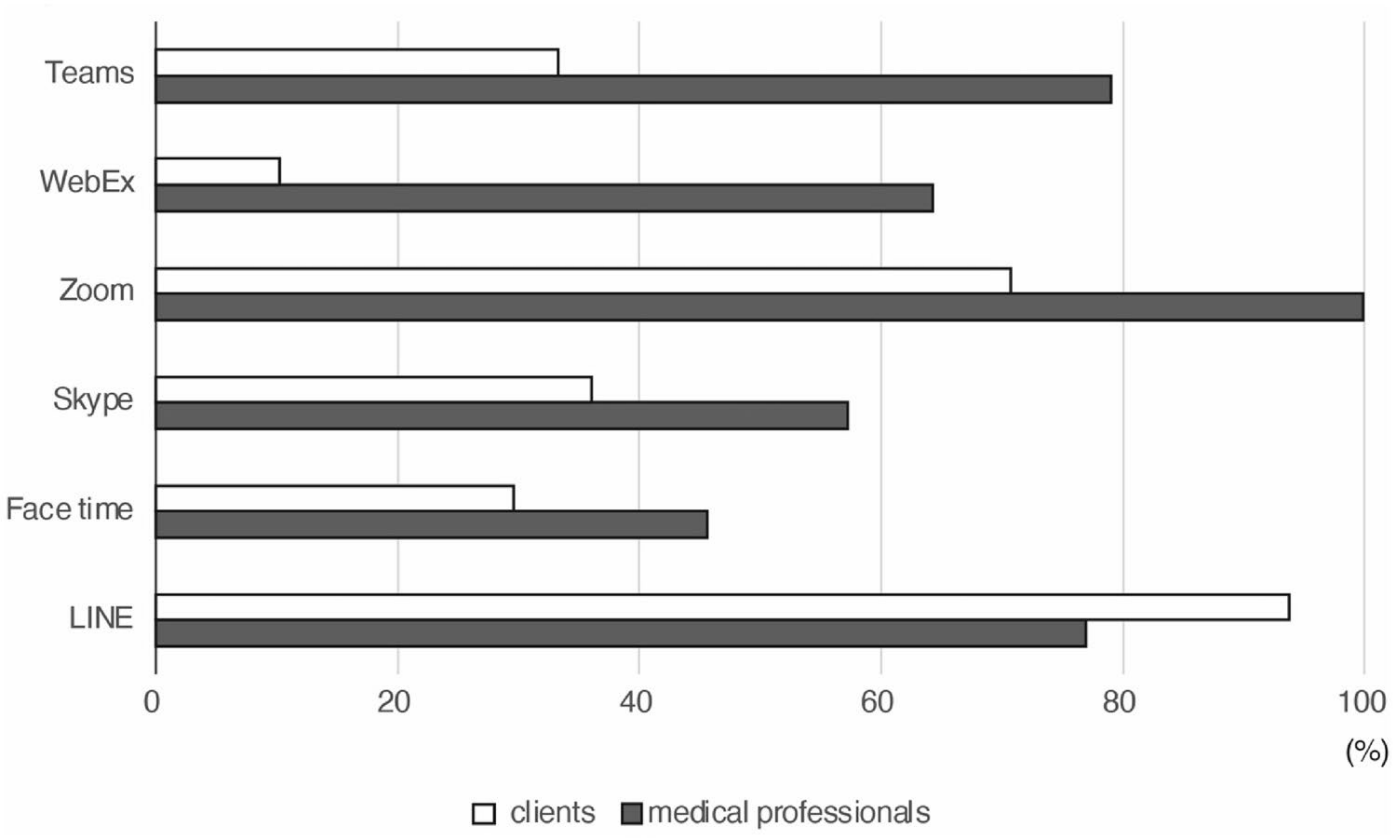
Proportion of clients and medical professionals who had used each web conferencing tool. The most familiar tool for clients was the LINE messaging app, followed by Zoom. Less than 40% of the respondents had used other tools. For medical professionals, the most familiar tool was Zoom, followed by Teams. Just over half of the respondents had used other tools

Of 425 clients who responded about device usage, nearly all currently used a smartphone (Fig. 4a). While 402 (90.7%) had used a desktop or laptop computer or a tablet, only 364 (82.2%) could operate these devices at home (Fig. 4b). In contrast, almost all clients could operate a smartphone independently at home.

**Fig. 5:**
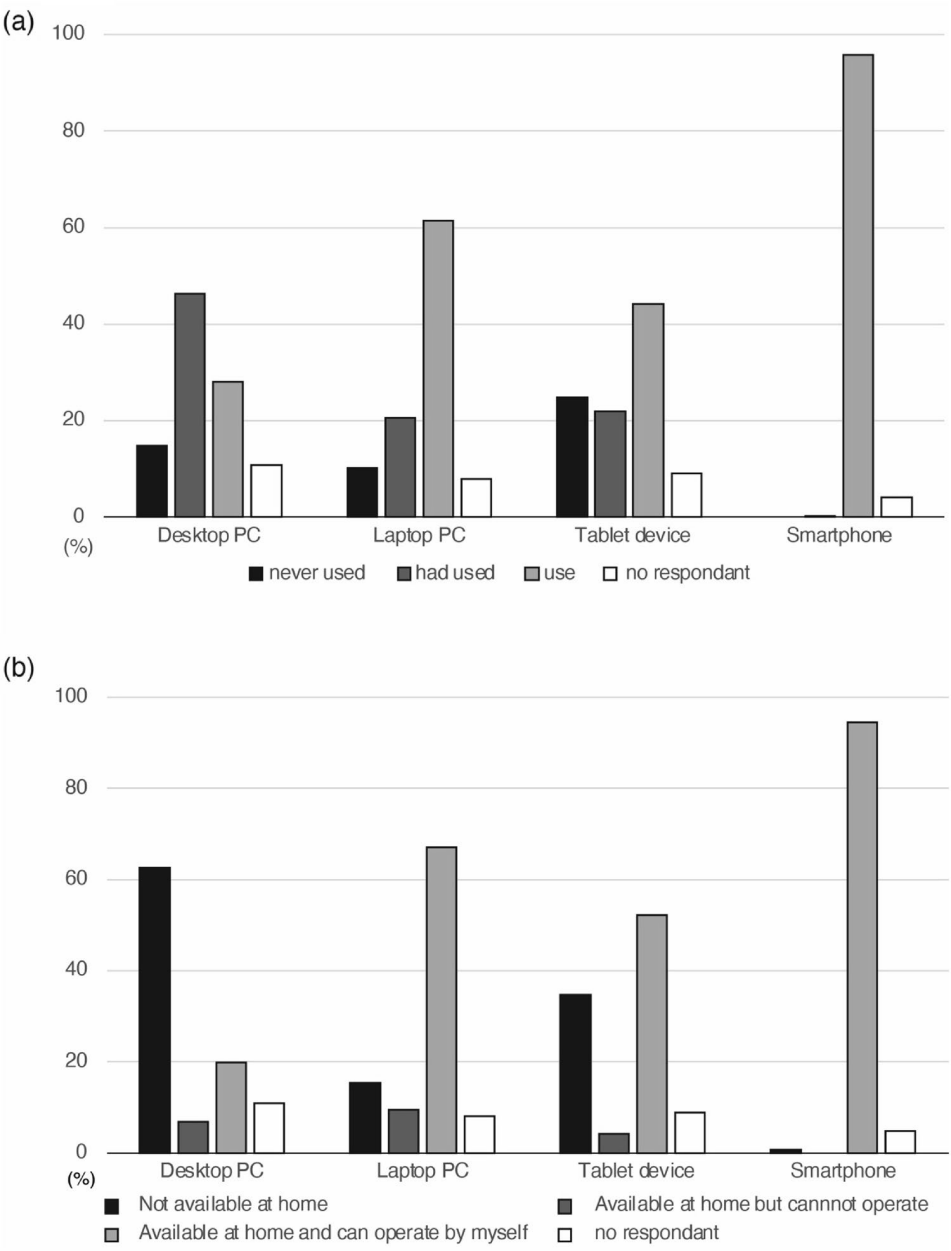
Clients’ experience with and current use of computer devices. **a** Most clients had used a smartphone, while 9.3% had never used a desktop PC, laptop PC, or tablet. **b** Most clients had and could operate a smartphone independently, while 82.2% had and could operate a laptop PC, desktop PC, or tablet. Devices other than smartphones were not available at home for 12.0%

Regarding video conferencing tools, 850 (99.1%) of medical professionals answered, “know about them”, compared to 405 (92.0%) of clients. Among those answered “know about them”, 9 (1.1%) medical professionals and 49 (12.1%) clients answered “have never used,” 43 (5.1%) and 24 (5.9%) answered “can use them with support from others,” and 798 (93.9%) and 332 (82.0%) answered “use them,” respectively, indicating that clients had less experience with these tools than the medical professionals did. The most used tool among clients was the LINE messaging app (330/352, 93.8%), followed by Zoom (249/352, 70.2%) (Fig. 5). Almost all medical professionals indicated they had used Zoom and, except for LINE, they were more familiar with all of the tools than the clients were.

## Discussion

In this study, we conducted two questionnaire surveys: one for medical professionals who offered genetic care and one for clients who received such care. The findings provided broad insight into the feasibility and challenges of implementing online genetic care in Japan from both sides’ perspectives. At the five hospitals where this survey was conducted, few online medical services have been offered, and most of the responding clients evidently had no experience with such services. Our findings that only 14.1% of responding medical professionals have experience with online genetic care aligns with the report of low overall online medical care adoption in Japan [14]. Therefore, this study primarily explored perceptions of individuals who have not yet experienced online genetic care, identifying key psychological and operational hurdles to its future adoption.

We uncovered a notable discrepancy between what client’s value and what medical professionals believe they value in selecting a medical institution for genetic care. Clients prioritized access to “specialty hospitals”, a need that online genetic care is uniquely positioned to address given the regional maldistribution of genetic care specialists in Japan. Medical professionals, however, assumed clients would place a higher value on “location where family members could also easily visit”. This may reflect the medical professionals’ subconscious awareness that genetic information affects blood relatives and that clients would like their family members to participate in GC sessions. These assumptions are consistent with the result that, as possible advantages of online GC, the clients more highly value “being able to visit their hospital of choice” and the medical professionals more highly value “enabling blood relatives to participate in the counseling”. These discrepancies suggest that clients may perceive genetic diagnosis as solely applicable to themselves, potentially failing to understand that it can affect their entire family. Further public awareness campaigns are needed to better inform the general population that appropriate surveillance and risk-reducing surgery can improve the prognosis of hereditary cancer syndrome carriers. We also demonstrated that clients’ expectations of genetic care vary depending on their location. While there were no differences in age, gender, frequency of internet use, or experience with web conferencing tools between clients visiting urban and rural hospitals, a clear difference was observed in travel time. Urban clients prioritized greater convenience, despite having shorter travel times, while rural clients tended to value face-to-face communication. These results indicate a need to develop genetic care systems tailored to the location of facilities.

Unlike previous study that linked telemedicine use to younger age and female gender among medical professionals [17], our study found no such association. Instead, differences in professions and years engaged in genetic care had the following effects. CGCs or CNSs had a higher percentage of experience, which may be because, unlike medical doctors, many of these professionals are involved in managing various genetic disorders. While confidence in hosting a web conference and years engaged in genetic practice were associated with past use of online genetic care, they did not predict future willingness to adopt it. These findings suggest a broad consensus among medical professionals on the utility of online genetic care and also highlight the need to develop a system that is usable irrespective of individual internet literacy. Apprehension remains a key barrier, medical professionals with greater concerns were less experienced and less willing to use online genetic care, underscoring the importance of role-playing of online GC and other simulated training as well as opportunities to observe actual online GC to promote its use.

Consistent with previous research [16], younger age was associated with receptivity to online genetic care. The current study further found that more internet usage and lesser concerns about online genetic care were positively associated with receptivity to such care. Clients showed lower anxiety about online genetic care than medical professionals did in all categories, suggesting the clients may more readily accept such care. One potential application of online genetic care in cancer treatment would be providing genetic information and cascade testing directly to at-risk relatives. Consequent appropriate surveillance and risk-reducing surgery could reduce future cancer risk and enable early cancer detection. However, 10.9% of the respondents anticipated that their understanding would be insufficient in an online setting. Higher age, infrequent internet usage, and greater concerns about online GC were identified as risk factors. One effective approach to address concerns about online communication would be to combine online sessions with supplementary face-to-face explanations and psychological support from another healthcare provider.

The digital divide remains a practical challenge. While personal internet usage in Japan is high, it declines significantly after age 60 [18]. In this study, the rate of Internet use at home was 92.4% for those in their 60 s, declining to 73.5% for those in their 70 s and older. This aligns with our finding that 62.4% of clients answered “it depends on the situation” as to whether they would recommend online GC to their relatives, concerns about their relatives’ ability to use the necessary technology. The high penetration of smartphones and the familiarity of clients with apps like LINE suggest that a successful system should be simple, perhaps operable with a single touch. However, the small screen of a smartphone makes it difficult to read a client’s emotional state, a critical aspect of GC. One potential solution could be establishing dedicated online genetic care booths equipped with large screens, high-speed internet connections, and high-precision microphones and cameras, and staffed by support personnel. These booths could be located in public health centers, post offices, and public libraries.

Several institutional barriers must also be addressed. Medical professionals expressed significant concerns about internet security and unauthorized recordings. Furthermore, many medical institutions may lack the necessary systems and infrastructure to offer online genetic care. Future research should investigate these institutional challenges and evaluate the effectiveness of proposed solutions.

This study has several limitations. First, the voluntary nature of the surveys may have introduced selection bias, as individuals already interested in online genetic care may have been more likely to respond, leading to overestimation of online GC acceptance. Second, most clients who responded the survey were assumed to have no prior experience using online medical services and may not have had a clear idea of whether or not they would encourage online genetic care to their relatives. Third, we did not collect the information about the history of cancer among clients, which would influence online GC acceptance. Finally, medical professionals’ experience and willingness may be influenced by the policies and available infrastructure at their institutions, which were not assessed in this study.

In conclusion, our survey illuminated the current state of online genetic care utilization in Japan and identified key challenges to its widespread adoption. To provide appropriate and satisfactory genetic care to all client, irrespective of their location, it is imperative to educate both patients and medical professionals, and develop an online genetic care system that is secure, accessible, and easily usable for all relevant parties.

## Data Availability

The datasets generated during and analyzed during this study are available from the corresponding author on reasonable request.
